# The Pathogenic Role of *Actinomyces* spp. and Related Organisms in Genitourinary Infections: Discoveries in the New, Modern Diagnostic Era

**DOI:** 10.3390/antibiotics9080524

**Published:** 2020-08-17

**Authors:** Márió Gajdács, Edit Urbán

**Affiliations:** 1 Department of Pharmacodynamics and Biopharmacy, Faculty of Pharmacy, University of Szeged, 6720 Szeged, Hungary; gajdacs.mario@pharm.u-szeged.hu; 2 Institute of Medical Microbiology, Faculty of Medicine, Semmelweis University, 1089 Budapest, Hungary; 3 Institute of Translational Medicine, Faculty of Medicine, University of Pécs, 7624 Pécs, Hungary

**Keywords:** *Actinomyces*, *Actinomyces*-like organisms, *Actinotignum*, anaerobe, MALDI-TOF MS, sequencing, genitourinary, taxonomy

## Abstract

Actinomycosis is a chronic, suppurative, granulomatous infectious disease, caused by different species of *Actinomyces* bacteria. To date, 26 validly published *Actinomyces* species have been described as part of a normal human microbiota or from human clinical specimens. Due to the rapid spread of new, modern diagnostic procedures, 13 of 26 of these species have been described in this century and the *Actinomycetaceae* family has undergone several taxonomic revisions, including the introduction of many novel species termed *Actinomyces*-like organisms (ALOs). There is scarce data available on the role of these novel bacterial species in various infectious processes in human medicine. The aim of this review is to provide a comprehensive overview of *Actinomyces* and closely related organisms involved in human diseases—with a special focus on newly described species—in particular their role in genitourinary tract infections in females and males.

## 1. Introduction, Taxonomy of *Actinomyces* and Closely Related Species

Actinomycosis is a chronic, suppurative, granulomatous infectious disease, caused by different species of *Actinomyces* bacteria [1,2]. The disease affecting animals was first described by Harz in 1878 (corresponding to *Actinomyces bovis*) [3], while the disease of human origin was first reported by Israel around the same time [4]. The suspected causative agent in these human infections, originally termed *Streptothrix israelii* (currently *Actinomyces israelii*) was correctly identified later in 1896 by Kruse [5]. A long time passed before the next species of the genus was described in 1951: *Actinomyces naeslundii* was obtained from the oral samples of the mouths of patients implicated with actinomycotic lesions [6], while two other species were descovered only a few years later: *A. odontolyticus* from deep carious dentine (1958) and *Actinomyces viscosus* (first described as *Odontomyces viscosus*) from the periodontal plaque in hamsters (1969) [7,8,9]. It is interesting to note that—so far—all *Actinomyces* species were isolated from the microbiota associated with humans and animals (exclusively mammals), while actinomycetes are usually soil dwelling bacteria, ubiquitous in the environment; e.g., *A. israelii* may be detected in decaying organic matters [10] and Rao et al. isolated *Actinomyces naturae* for the first time from chlorinated, solvent-contaminated groundwater in 2012 [11].

**Figure 1:**
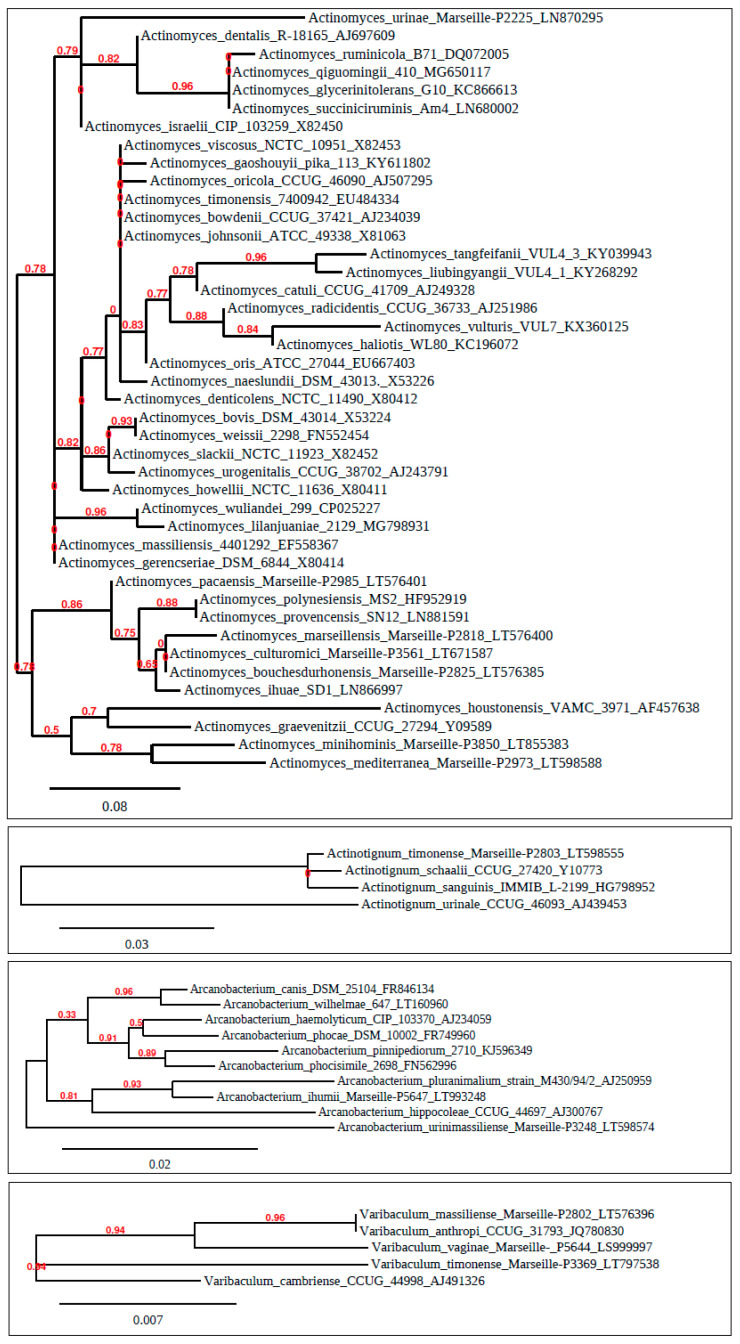
Phylogenetic analysis of bacterial genera discussed in this paper (namely *Actinomyces*, *Actinotignum*, *Arcanobacterium,* and *Varibaculum*) including both pathogenic and non-pathogenic strains. Bacterial 16S rRNA gene sequences were retrieved from the LPSN (List of Prokaryotic Names with Standing in Nomenclature) database [39], while analysing phylogenetic relationships were analysed using Phylogeny.fr in a PhyML and BLAST-EXPLORER environment [40,41].

According to the recent taxonomic classification, the clinically relevant and heterogenous group of anaerobic, non-spore forming, non-acid-fast, Gram-positive bacilli may be divided into two phyla: Actinobacteria and Firmicutes [12]. The classification of *Actinomyces* spp. (which was previously primarily based on phenotypic characteristics) has been revised recently, due to advances in microbial taxonomy and the use of genotypic methods, such as comparative 16S ribosomal RNA (rRNA) gene sequencing [12,13,14]. Since the introduction of 16S rRNA gene sequencing, the number of individual *Actinomyces* species described has increased considerably [1,12,13,14]. Taxonomically, the genus *Actinomyces* belongs to the Actinomycetales order of the Actinobacteria phylum (characterized by high guanine-cytosine (G+C) content in their genome, e.g., 55%–68% in case of the *Actinomyces* genus (Figure 1)) [15]. The genomic evolution of the members of the family *Actinomycetaceae* is very dynamic, some studies observed that mutations or loss of genes in the species in the family contribute to changes in the microbial behavior and habitat preferences [16]. The genera of *Mobiluncus* and *Actinomyces* form a monophyletic clade in a phylogenetic tree constructed based on RpoB, RpoC and DNA gyrase B protein sequences [17,18]. The existence of this clade is also strongly supported by a conserved signature indel, consisting of a three-amino-acid insertion in the isoleucine-tRNA synthetase found only in the species of the genera *Actinomyces* and *Mobiluncus* [16,17,18]. *Actinomyces* is one of six genera within the family *Actinomycetaceae*, and currently has 53 child taxa with validly published names, including synonyms [15,16,17,18,19]. The first deposited type strain of the *Actinomyces* species is *A. bovis* (as previously mentioned) [3,19]. There are three leading clusters by a phylogenetic tree based on 16S rRNA gene sequence comparisons, althought not all of them have been strengthened by the bootstrap analysis [16,17,18]. The majority of species belong to the cluster that includes *A. bovis*, while the other two clusters, which include *Actinomyces neuii* and *Actinomyces hordeovulneris* should be investigated further to determine if they warrant proposal as novel genera [16,17,18]. The description of novel bacterial species was further facilitated by the newfound interest in the characterization of the human microbiome [20]. According to culture-independent studies, there have been a number of new *Actinomyces* species proposed; e.g., some of them have been described as a part of the Human Oral Microbiome Database (HOMD), listing 18 species-level *Actinomyces* taxa, which are validly not yet published or currently unnamed [21,22].

In addition to *Actinomyces* spp., the Actinomycetales order includes other important genera, such as *Actinobaculum*, *Arcanobacterium*, *Cellulomonas*, *Mobiluncus,* and *Varibaculum* [23]; these genera are the most similar, as they share several phenotypic characteristics (e.g., aerotolerance, pleomorphic morphology, non-acid fast staining), distinct from the other members of the order [24]. Several former members have been reclassified; taxonomic reassessment of members of the *Actinomyces* genus has led to some species being assigned to other genera, such as *Actinobaculum suis* (previously *Actinomyces suis*) [25], *Cellulomonas humilata* (formerly *Actinomyces humiferus*) [26], *Trueperella bernardiae* (formerly *Actinomyces bernardiae* and later, *Arcanobacterium bernardiae*) [27], and *Trueperella pyogenes* (formerly *Actinomyces pyogenes* and later, *Arcanobacterium pyogenes*) (Figure 1) [28]. Three other human and one animal *Actinomyces*-like organisms (ALOs) were also described as novel species [29]; these were first described in 1997, when a taxonomic division occurred, separating them from *Actinomyces* spp.: *Actinotignum* (formerly: *Actinobaculum*) *schaalii* [30], *Actinobaculum massiliae* (later *A. massiliense*) [31], *Actinotignum* (formerly: *Actinobaculum*) *urinale* [32]), and *Actinobaculum suis* [25]. The species of *A. schaalii* and *A. urinale* have been reclassified and assigned as a brand-new genus *Actinotignum*, in addition to a strain from human blood, that has been characterized and described as *Actinotignum sanguinis* by Yassin et al. in 2014 [33]. As of 2015, the former genus *Actinobaculum* has been officially split into two different genera, *Actinotignum* and *Actinobaculum* [34]. In one earlier study from the Anaerobic Reference Unit (ARU) of Cardiff, UK, a group of ALOs isolated from human infections were found, which reporesented a novel genus of *Varibaculum*, within the family *Actinomycetaceae* [35]. Some other so called “novel species” (or *species novum*, *spec. nov.*) were “*Actinomyces lingnae*” and “*Actinomyces houstonensis*” were proposed in this report [36], but due to the fact that the descriptions of the species were incomplete and the strains have not been deposited within international culture collections, the proposals have not been validated yet.

In a recent review, 26 individual *Actinomyces* species were listed, which were known to be implicated in human infections (see Table 1) [37], but it would be safe to assume that more than these 26 species may have had a role in clinically relevant infectious pathologies. The number of these reported species may be a reported estimation only, as clinical isolates were frequently misidentified before the introduction of current diagnostic technologies (e.g., DNA-DNA hybridization, polymerase chain reaction (PCR), matrix-assisted laser desorption/ionization time-of-flight mass spectrometry (MALDI-TOF MS) and whole-genome/next-generation sequencing (WGS/NGS) [1,12,37,38]. According to the data of the literature a wide range of *Actinomyces* species has been reported with increasing frequency nowadays as causes of infection at various body sites [1,12,37].

## 2. Microbiological Characteristics

**Figure 2:**
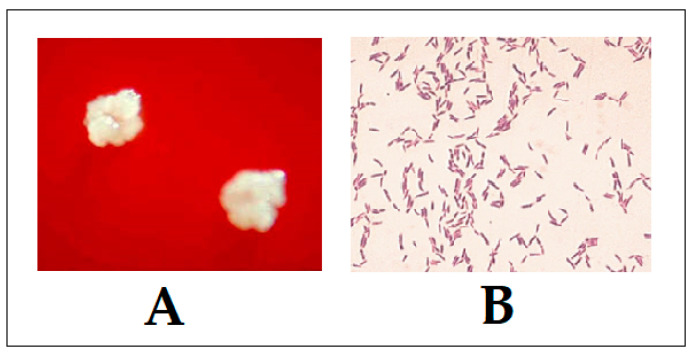
Typical colony morphology (**A**) and Gram-staining (**B**) of *A. bovis.*

The name of the genus originates from the greek words: *aktinos* (ray) and *mykes* (fungus), which corresponds to the radial arrangement of the bacterial filaments (resembling hyphae) and the possession of reproductive asexual spores, typical for filamentous fungi [42]. This is often misleading for clinicians, designating these bacteria as “rayfungus” [43]. The members of genera require varying degrees of anaerobiosis (i.e., they grow best under anaerobic conditions), but the majority of medically relevant strains are aerotolerant (with the exception of *Actinomyces meyeri* and *A. israeli* being both obligate anaerobic), saprophytic, non-spore-forming branching, filamentous, Gram-positive rods [1,12,44]. Several other genera, including *Frankia, Nocardia,* and *Streptomyces* are frequently (and incorrectly) referred to as the “actinomycetes” group in clinical practice, due to their similar radiating or branching morphologies [45]. The traditional bacteriological cultivation and identification of *Actinomyces* strains from a sterile site or infected region may confirm the diagnosis of actinomycosis, but the isolation and identification of these bacteria only occurs in some cases [46,47]. The success of culture is low, because of the growth inhibition of *Actinomyces* by other microorganisms, inadequate culture conditions or inadequate (i.e., short-term) incubation [48,49]. Throughout sample handling and culture, strict anaerobic processing (rapid transport in an anaerobic transport medium), anaerobic growth conditions should be used for primary cultivation and prolonged culture on appropriate media (growth of *Actinomyces* is slow; at least 5 days are needed for colonies to form on solid media and may even take up to 15–20 days) and appropriate atmosphere is performed [50]. Thus, incubation of at least 14 days is recommended before a conclusion of a negative culture [1,37,51]. The optimal temperature for growth of *Actinomyces*, namely 30 °C to 37 °C, suggests a primary human and animal source of bacteria [12]. In routine clinical practice, samples containing *Actinomyces* strains may be cultured on anaerobic blood agar (ABA) media complemented with sheep blood at 37 °C; other enriched media can also be used for the isolation of *Actinomyces* spp.: brain heart infusion (BHI) broth and *Brucella* blood agar (BBA) supplemented with hemin and Vitamin K_1_ [52]. The use of semi-selective media (such as phenylethyl alcohol, mupirocin-metronidazole blood agar or *Actinomyces* isolation agar) may increase isolation rates by inhibiting overgrowth of co-organisms, especially members of *Enterobacteriaceae* [53]. *Actinomyces* strains can be initially suspected by typical colony morphology and Gram-staining results. Colony morphology varies from “molar tooth”, spider- or cobweb-like appearance to “breadcrumb” colonies on solid media [54]. While most of the colonies of *Actinomyces* isolates are white, different extents of pigment production may be observed in some species when had prolonged incubation period on blood agar (Figure 2) [54].

**Figure 3:**
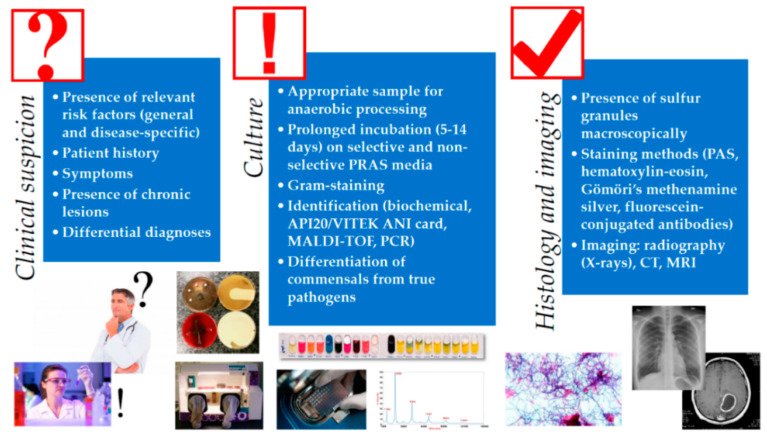
Diagnostic algorithm for the diagnosis of *Actinomyces* infections (reproduced with permission from [1]). Abbreviations: PRAS: pre-reduced, anaerobically sterilized; MALDI-TOF: matrix-assisted laser desorption-ionization time-of-flight mass spectrometry; PCR: polymerase chain reaction; PAS: periodic acid–Schiff stain; CT: computer tomography; MRI: magnetic resonance imaging.

The identification and differentiation of the anaerobic Gram-positive rods and species belonging to the genus *Actinomyces* may cause major troubles for clinical microbiology laboratories in terms of labor and the time-consuming nature of the process when conventional (biochemical reaction-based) methods are used [55]. Identification was traditionally based on phenotypic tests, which were based on the detection of the presence of various enzymes (e.g., oxidase, urease, catalase, fermentation of sugars, and indol production) and commercial biochemical kits; however, these tests often lead to misidentification on the species- and even genus-level of strains [56]. Furthermore, the databases of actually available commercial identification kits are limited and they do not adequately identify most of the newer species (Figure 3). For example, phenotypic identification of *Actinomyces* by VITEK^®^ 2 240 ANC ID Card (bioMérieux, Marcy-l’Étoile, France) was limited to seven *Actinomyces* species and *A. schaalii* [57]. Currently, mostly molecular diagnostic methods serve as the reference for the identification of these microorganisms, besides 16S rRNA sequencing [58]. Appropriate identification methods also include 16S ribosomal DNA restriction analysis and polymerase chain reaction (PCR) with specific primers; these maybe used for direct detection of *Actinomyces* both from clinical specimens and for identification from isolated colonies [59]. Nowadays, the MALDI-TOF MS method is a quick and accurate tool for the identification of anaerobic bacteria, including *Actinomyces* species (Figure 3) [60,61]. The MALDI-TOF method allows excellent, rapid and accurate identification at the genus level, but species identification remains uncertain and largely depends on the mass spectrometry systems and the available database, but improvement of this method is needed for a definitive identification of *Actinomyces* spp. [62,63,64]. This it will also likely facilitate the increase in knowledge about the natural habitat and therefore the relative pathogenicity of these various species [65].

## 3. *Actinomyces* Species as Members of the Human Microbiota

Nowadays, it is well-known that actinomycosis is an endogenous infection of human or animal origin [66]. At the phylum level, Actinobacteria are frequent colonizers of most ecological niches of the human and animal bodies [67]. *Actinomyces* spp. are predominantly found in the human oral cavity. In addition to the mouth, these organisms may also be found in different anatomial regions, colonizing the upper respiratory tract, gastrointestinal tract, the female genitourinary tract, and very rarely, the skin [67,68]. They are not normally always present on the surface of the skin, due to the secretion of various inhibitory molecules (fatty acids, antibacterial peptides) by other bacteria (which are normal members of the skin flora) [69]. If the mucosal barrier is disrupted, mucosal-resident *Actinomyces* species gain access to deeper tissues via trauma, surgical procedures, or foreign bodies, and inside these tissues, the bacteria form masses consisting of aggregates of branching, filamentous bacilli [70]. Little is known about the virulence factors of pathogenic *Actinomyces* strains by which they cause infections [71]. Most infections with *Actinomyces* spp. are polymicrobial and members of the *Streptococcus* genus are the most commonly associated organisms [72]. They act synergistically by inhibiting host defence mechanisms and reducing oxygen tension in the affected tissue, which enhances growth of *Actinomyces* spp. [73].

Currently, some evidence has accumulated on how *Actinomyces* species might play a role in biofilm formation, e.g., in the oral cavity [74]. The hydrolysis of urea by the urease enzymes of members of oral microbiota might have a major impact on oral microbial ecology and is closely involved in the maintenance of oral health and/or diseases. In many anatomical locations, such as the oral cavity and female genital tract, the excess carbohydrate availability and neutral pH environments could promote urease expression of *Actinomyces* in biofilms, but only neutral pH environments could up-regulate the *ureC* gene expression (as pH regulates *ureC* gene expression at a transcriptional level) [75,76]. Most of the investigated *Actinomyces* strains are able to produce cell-associated/extracellular polymers, such as dextran, levan, glycogen, and *N*-acetylglucosamine-rich slime polysaccharides, therefore, the cells can attach to different (own or foreign) surfaces [77]. In addition, *Actinomyces* spp. possess different fimbriae, involved in their pathogenicity: type 1 fimbriae help the organism to adhere to salivary proline-rich proteins that coat the tooth surface, while type 2 fimbriae mediate the receptor-dependent coaggregation between *Actinomyces* and streptococci or the host cells during the development of oral biofilms [72,78]. By using different clinical and type strains, some detailed molecular and biochemical studies have been carried out to investigate the genes that encode the structural protein subunits of type 1 and type 2 fimbriae [78,79]. There identified that the FimP and FimA fimbriae require a sortase-like genes to assemble type 1 and 2 fimbriae, respectively, as both contain a pilin motif and an E-box, which are common features of Gram-positive bacterial major pilin subunits [78,79,80].

## 4. Actinomycosis of the Genitourinary Tract

### 4.1. Infections of the Female Genital Tract

Actinomycosis infections are relatively rare (at least in Western populations, with an estimated prevalence of 1/300,000 persons) [1,12,37]. However, actinomycosis of the female genitourinary tract is the second most frequent clinical mainfestation of this disease [81]. The most common form of genitourinary tract actinomycosis is pelvic actinomycosis in young, fertile women using intrauterine contraceptive devices (IUDs), but nowadays, according to the introduction of modern microbiological techniques, some other clinical presentations have also been described, such as primary bladder actinomycosis and testicular actinomycosis [82,83]. Different strains of *Actinomyces* species found normally in the human (female) urogenital tract include: *A. meyeri, A. neuii, Actinomyces radingae, Actinomyces turicensis,* and *Actinomyces urogenitalis* [84,85,86,87,88]. Colonization of the female genital tract by these *Actinomyces* spp. is greatly promoted by the long-term use of IUD, as IUDs have a permanent irritating effect of the endothelium by causing erosion and subsequent trauma, which may facilitate the invasion of bacteria [89]. When IUD-associated actinomycosis is established, abscess formation in the genital tract is frequent, and the spread of bacteria from pelvic sites to the abdominal region or vice versa can lead to abdomino-pelvic actinomycosis, creating dense adhesions with contiguous structures, including the small bowel, causing fibrosis, fistulas, and peritonitis [90,91]. Formerly *A. israelii* was one of the most common species involved in pelvic actinomycosis, but the pathogenic role of many novel species has been recently described [85,86,87,88]. According to the review of Fiorino in 1996, the recovery of <100 actinomycotic specimens between 1926 and 1995—most of those being tubo-ovarian abscesses—indicated that the risk of pelvic actinomycosis in relation to the use of IUDs is very low [92]. At the beginning of the molecular diagnostic era, culturing data from a reference laboratory in Germany, published by Schaal et al. showed that that *A. israelii* was the most frequent (61%) isolate from IUDs and cervical secretions in 1992, while *A. viscosus* was the second most common species, with a 18.3% share; in contrast, *Actinomyces gerencseriae*, *A. naeslundii* and *A. odontolyticus* were identified in only 4.9% 4.9% and 3.7% out of the 82 specimens, respectively [93]. However, research involving new molecular diagnostic methods has further nuanced these results [94]. 113 *Actinomyces* isolates from clinical material associated with IUDs were sent for processing in the ARU in Cardiff, UK to be identified on the species-level with the ARDRA (amplified 16S rDNA restriction analysis) method [95,96]. One-third of strains were identified as *A. israelii* and *A. turicensis*, the “*A. naeslundii-A. viscosus*” complex, *A. odontolyticus* and *A. gerencseriae* were identified as the principal species but *Actinomyces cardiffensis* and *Actinomyces funkei* were also common [95,96]. It is curious that in this study, 11 strains have originated from the pelvis and 37 strains originated from samples from the vagina/penis were obtained, and among isolates originating from PID and pelvis samples, *A. turicensis* was the most common species (26/37) [97]. The same study group identified 8 previously unknown isolates from human clinical sources. Three of them were recovered from IUDs: one from an IUD which was in situ for 7 years in a 35-year-old female, while two others from IUDs of 26- and 37-year-old females in 2000 and 1994, respectively); the isolate identified was as a new species, namely *A. cardiffensis* sp. nov. [98]. In their study, Woo et al. [99] isolated an anaerobic Gram-positive non-sporulating bacterium from an IUD of a 36-year-old woman with pyosalpinx in 2002. The 16S ribosomal RNA gene of the strain was amplified and sequenced, and it was found that the strain is a typical oral species: *A. odontolyticus*. Later on, the same authors reported a case of a 36-year old Chinese woman, with an IUD inserted for more than one year and pelvic inflammatory disease (PID) when bilateral laparoscopic salpingotomy was performed. Bacterial strains were isolated from the pus of both ovarian tubes and the IUD was removed; a brand-new species (*Actinomyces hongkongensis*) was proposed based on the 16S ribosomal RNA (rRNA) gene sequencing data [100]. Another case of pelvic actinomycosis due to *A. hongkongensis* was reported later by Flynn et al. [101] from Canada, resulting from a routine gynecologic procedure, hysterectomy and salpingectomy. They desribed the case of a 47-year-old, immunocompetent woman who presented at the hospital with pelvic pain and fever 13 days after a total abdominal hysterectomy and salpingectomy for menorrhagia and hydrosalpinx. After the operation, a bilocular abscess developed immediately above the vaginal vault and a strain cultured from the fluid aspirate was identified as *A. hongkongensis* by 16S rRNA gene sequencing [101].

*A. turicensis* appears to play a role in IUD-associated pelvic actinomycosis: Ong et al. [102] published a very instructive and unusual case of *A. turicensis* infection mimicking an ovarian tumour. Some gynecological procedures may cause an individual to have complications associated with *Actinomyces* bacteria; Van Hoecke et al. [103] reported a secondary *A. urogenitalis* bacteremia in a non-IUD user who had a tubo-ovarian abscess following transvaginal oocyte retrieval procedure. *A. urogenitalis* was described as a novel species in 2000 by Nikolaitchouk et al. [104], based upon the characterization of three isolates recovered from human clinical specimens from the urogenital tract (from the urine of a of a 70-year-old woman, from the urethra of a 44-year-old patient and from the vaginal secretion of a 33-year-old woman, who had abnormal discharge, possibly due to the use of an intrauterine device for 7 years; the recovery rate was ~10^7^ colony-forming units [CFU]/mL). Since then, it has become clear that *A. urogenitalis* is member of the normal flora of the human vagina. Colonization and recovery with high concentrations of *A. urogenitalis* from vaginal samples may occur in cases of bacterial vaginosis, but the role of these bacteria in the pathogenesis is unclear. *A. turicensis* can be detected in a variety of infections of the female genital tract, such as adnexitis, endometritis, cervicitis, vaginitis, and vulvitis [105]. In the study of Sabbe et al. [106], *n* = 294 ALOs were isolated over a 7-year period and these strains investigated by DNA sequence analysis. As a general rule. the organs of the genital tract were apparently the sites most frequently infected with ALOs (with *A. turicensis* in particular), while in this report *A. radingae* and *Actinomyces europaeus* were not detected in the genitourinary tracts of either males or females. They found *A. turicensis* isolates in female patients with adnexitis, endometritis, cervicitis, vaginitis, and vulvitis, and in a few instances, the isolate was found in pure culture [106]. Their pathogenic role was also described in the formation of various genital abscesses, (including vulvar or perineal abscesses and Bartholin’s abscess). leukorrhea, control cultures (e.g., after gonorrhea therapy in patients with IUDs or in pregnant women with ruptured membranes for more than 24 h). Suprisingly, three of the patients with vulvitis and seven of the patients with vaginitis or leukorrhea caused by *A. turicensis* were prepubertal [106]. Funke and von Graevenitz [107] isolated a newly proposed species: *A. neuii* in infected amniotic fluid of pregnant women. They discussed that in their cases presented, this species did not cause the typical presentation of actinomycosis. Some publications recorded the role of *A. neuii* in severe cases of chorioamnionitis, leading to sepsis of the neonate: the first such report of sepsis caused by *A. neuii* in a preterm neonate parallel with the presence of *A. neuii* in the maternal genitourinary tract was published by Mann et al. in 2002 [108]. The *A. neuii* strain was found not only in the vaginal smear of the mother and in the amniotic fluid, but also in the child’s blood culture. Later, de Velasco-Sada et al. [109] described a case of neonatal sepsis, secondary to chorioamnionitis by *A. neuii* in a 25-week pregnant woman. Wright et al. presented a case of necrotizing funisitis (inflammation of the connective tissue of the umbilical cord) in a 24-year-old pregnant woman, who experienced preterm birth at 31th weeks of gestation. Examination of the placenta revealed severe chorioamnionitis and necrotizing funisitis with *A. meyeri* identified as the causative agent; large numbers of Gram-positive filamentous branching organisms could be found on the surface of the cord and within Wharton jelly [110].

### 4.2. Infections of the Male Genital Tract

Actinomycosis of the penis is an extremely rare condition, the real definitive diagnosis is cutaneous actinomycosis and most cases present an association with the pilonidal sinus of the penis [111,112]. Min et al. reported a very rare case, with an unusual site of primary penile cutaneous actinomycosis clinically presenting as an epidermal cyst. In infections of the genital tract in males, *A. neuii* has mostly been detected in prostatitis cases: in the abovementioned article by Funke and von Graevenitz [107], *A. neuii* strains were repeatedly isolated in pure culture of the ejaculate from two male patients with prostatitis, in addition to two other patients with urinary tract infections, where *A. neuii* was detected in pure culture in more than 10^6^ CFU/mL. *A. turicensis* has rarely been detected in balanitis, penile abscess and prostatitis cases: this pathogen was found by Sabbe et al. [106] in eight patients with mild to severe balanitis, including a patient with a penile ulcer and a concurrent *Chlamydia trachomatis* infection. In four other patients, non-hemolytic streptococci were also found as co-isolates, while in three instances, *A. turicensis* was the only potential pathogen present. All investigated patients except for a 2-year-old boy were sexually active adults. Three isolates from patients with balanitis could not be identified correctly on the species level. In their study, urethritis was one of the most frequently occurring pathology in males. All six patients with urethritis from whom cultures were taken carried *A. turicensis*. One patient had a urethral ulcus; in the sample from this patient with ulcus, a mixture of various facultative and anaerobic bacteria was found, including *A. turicensis* together with *Neisseria gonorrhoeae* and *C. trachomatis*, respectively [106,113]. Other pathologies found in males consisted of a penile abscess and prostatitis, both of which occurred in older men (ages 61 and 85 years, respectively). In both patients *A. turicensis* was found at a high CFU/mL, only from samples from the patient with an abscess were other bacteria (anaerobes) cultured [114].

Quite rarely, but cases involving the male genital tract where ALOs played a pathogenic role have also been reported: Lara-Oya et al. [115] reported a very rare case of balanitis in a 85-year-old man, when the *A. schaalii* played pathogenic role and Jöhnk et al. [116] described a case of a 68-year-old male with phimosis caused by a severe lichen sclerosus et atrophicus (LSA) who suffered from *A. schaalii* urosepsis, with the same strain found in blood and urine. Ruiz-García et al. [117] reported a sole case of a 7-year-old boy that presented in the emergency department with inflammation of the penis, with a history of phimosis and balanopreputial adhesions. However, the patient did not have urinary symptoms or systemic manifestations, the most relevant finding of the history taking was that the patient had spent the weekend at the beach. Sample from an abundant yellow-green purulent exudate of foreskin and shaft of the penis yielded *A. schaalii* in pure culture.

### 4.3. Urinary Tract Actinomycosis

Until recently, the general dogma among clinicians and microbiologists was that the urine is normally sterile and that the normal flow of urine usually prevents bacteria from infecting the urinary tract [118,119]. In case of urinary retention, the abnormal flow of urine allows for bacteria to adhere to the epithelial lining and subsequently infect the urinary tract [120,121]. Thus, it is plausible that a similar mechanism contributes to the pathological processes associated with urinary tract actinomycosis [122]. The pathogenesis of primary bladder actinomycosis is unclear, but could be due to cryptic locations, and usually mimics bladder carcinoma; the lesions may invade adjacent organs such as the uterus or the sigmoid colon [123]. The correct differential diagnosis of primary bladder actinomycosis is very important, because of it might be protect unnecessary surgical resection for suspected tumor [124]. In both males and females, *A. turicensis* has been reported from clinical samples of urethritis and cystitis, *A. neuii* has been associated with urinary tract infection, *A. europaeus* with cystitis or purulent urethritis and as its name implies, *A. urogenitalis* strains were found in clinical samples from urethra and urine [12,37,106,125]. In these clinical samples, there are often some other aerobic and facultative bacteria present and only slightly elevated levels of leukocytes are observed in urine during urinalysis [12,37,106,125]. In addition to *Actinomyces* spp., other fastidious Gram-positive taxa has been recognized recently as etiological agents of urogenital infections, namely, *A. schaalii*, *Aerococcus urinae* and the much less known *Aerococcus sanguinicola* [126,127]. Interestingly, Rasmussen et al. [128] described a case when an *A. urinae* isolate was detected in parallel with *A. urogenitalis* in the urine of a patient who had been experiencing a prolonged period of urinary retention prior to the infection. The two *Aerococcus* species are well recognized etiological agents of urinary tract infection, both species have been associated with bacteremic/septicemic episodes, and in rare instances, *A. urinae* has also been identified as a causative agent of infective endocarditis [129]. Holmgaard et al. [125] described a case of bacteremia and urogenital infection with *A. urogenitalis* following prolonged urinary retention in an otherwise healthy sixty-year-old man, who had no prior urogenital medical history. *A. schaalii* was first described as a causative agent of human infections in 1997 by Lawson et al. [25], and since then, the uropathogenic role of the bacteria has been increasingly demonstrated, mainly causing UTIs in elderly individuals with underlying urological diseases [130]. Hall et al. in 2003 [131], from the ARU in the UK isolated, and then discovered, a hitherto undescribed new species from an anaerobic culture of urine of a female patient with pyuria, after repeated urine specimens from this patient yielded no bacterial growth under aerobic conditions. Based on both phenotypic and phylogenetic evidence (an almost complete 16S rRNA gene sequence was demonstrated), a novel species of the genus *Actinobaculum*, was proposed with the name *A. urinale* sp. nov. Sabbe et al. [106] isolated ALOs from the urine of 13 male and 12 female patients whom mostly had complains related to cystitis (except for three females, who had urethritis; in three females the same strain was found twice in a short time). Two patients had malignancies (one patient with carcinoma of the bladder and one patient with carcinoma of the prostate). The relatively newly discovered and more recently known *Actinotignum* species are present in the urine of healthy individuals, therefore these are probably part of the normal urogenital microbiota and sometimes associated with different urinary tract infections [132,133]. More and more reports recently have highlighted that the members of these species are causative agents of symptomatic UTIs and occasionally, they are also associated bacteremia, mostly in elderly patients [134,135]. The WGS of their genomes described the presence of some fimbrial genes encoding for attachment pili, which may be responsible for uroepithelial colonization, promoting the induction of UTIs in *A. schaalii* strains [136]. Olsen et al. [137] used PCR methods in their investigation and found different *Actinotignum* species on skin in the groin region of men and in the normal vaginal microbiota of women, however, they were not detected in the feces, which means that these species are unlikely to be a member of bowel microbiota, but a part of the urogenital flora. Based on literature data published so far (mainly focusing on case studies reporting on patients with some urological disorders and chronic prostatitis), it can be concluded that under some circumstances, epididymitis has been identidied as the source of *A. schaalii*, and this organism may be cause of “sterile” pyuria or epididymo-orchitis [132,133,134,135,136,137,138]. Elderly patients (mostly males) with recurrent sterile pyuria, or those with predisposing urogenital conditions—including benign prostate hyperplasia or prostate cancer, catheterization, bladder cancer, or neurologic bladder) and atypical genitourinary anatomy predisposing to reflux (such as urethral stenosis) or urinary incontinence, chronic renal failure, as well as with immunodepression—should have a urine sample sent for anaerobic culture if routine cultures are continuously sterile [132,133,134,135,136,137,138,139]. All of these studies or case reports suggest that *A. schaalii* is probably far more common in elderly- or middle-aged patients as previously thought, particularly if the clinical presentation is combination with leukocyturia and inconclusive bacteriological results from the routine microbiological testing [132,133,134,135,136,137,138,139,140]. Further investigations to detect *A. schaalii* in combination with the leukocyte count in the urine of vulnerable group patients suffering from urinary tract infection should be considered. Urine samples are usually only incubated in ambient air conditions for 24 hours or less, and the recognition of fastidious organisms, such as *A. urogenitalis*, *A. schaalii*, *A. urinae*, *A. sanguinicola*, may be missed altogether [121]. They are, however, most likely under-diagnosed in urine cultures, since most clinical microbiological laboratories routinely only use aerobic growth conditions.

There are only a few larger follow-up studies available in the literature: Bank et al. [141] from Denmark studied the prevalence of *A. schaalii* in urine samples using a specific real-time PCR examination of urine from patients with kidney stones in 2010. They have found *A. schaalii* in seven (29%) of the 24 leucocyte esterase-positive urines and in leucocyte esterase-negative urine samples, *A. schaalii* was found in 15% of the 52 samples. Later, they reported another study by the same RT-PCR method in a population of 252 patients, that included 177 hospitalized patients and 75 outpatients: a prevalence of 22% with *A. schaalii* with a bacterial load ranging from 10^4^ to >10^7^ CFU/mL was found in patients >60 years of age [142]. Lotte et al. [143] conducted a 3-year prospective study of *A. schaalii* related infections in a 1602-bed hospital in Nice, France. They found specimens positive for *A. schaalii* in 35/53 (66%) urine samples (40% of patients with positive urine samples had *A. schaalii*-related UTIs), 8/53 (15%) pus samples, 6/53 (11%) blood cultures and 4/53 (8%) spermocultures; most of these cultures (53%) were monobacterial. In addition to previous study reports [129,130,131,132,133,134,135,136], this study has also demonstrated that *A. schaalii* should be considered a true opportunistic uropathogen with the potential to spread and cause severe infections. Interestingly, the publication by Tschudin-Sutter et al. [144] in 2011, observed 27 patients from which *A. schaalii* was isolated in different clinical samples, but among these patients, *A. schaalii* was implicated in an invasive infection in 81.5% of cases (detected in blood cultures in 10 cases) and only half of them were associated with urinary tract infections. There are some other studies and especially unique case reports, when *A. schaalii* has mainly been considered as a pathogen in the context of UTIs corresponding to cases of treatment failure [132,133,134,135,136,137,138,139]. For the time being, there are still few studies that have been performed in the pediatric population, and these are also mainly rare case reports; therefore, in the pediatric population, *A. schaalii* infection is very limited. Using the same RT-PCR method as Bank et al. in their previous study, Andersen et al. studied 29 hospitalized children presenting with unspecific fever or symptoms of UTIs: *A. schaalii* was present in 33% of urine samples of these children [145,146]. Zimmermann et al. [147] in 2012 reported the case of an 8-month-old male patient, who suffered from myelomeningocele and neurogenic bladder and in contrast to previous reports in children with *A. schaalii* UTIs, the patient had no clinical symptoms. Sample from urine was cultivated and *A. schaalii* was detected from this urine culture in 10^4^  CFU/mL. The growth of small colonies of Gram-positive rods was observed after 48 h of incubation and an *A. schaalii* infection was confirmed by sequencing of the 16S rRNA gene. Their main conclusions were that *A. schaalii* is an emerging pathogen in patients of all ages, both in children and adults [132,133,134,135,136,137,138,139,140,141,142,143,144,145,146,147]. Previous colonization of urogenital tract (especially skin) and subsequent infection seem to be influenced by a number of factors in the particular age group of the patients. In infancy with a high suspicion of UTI who use diapers or young children who have known anatomical disorders or abnormalities related to their urogenital tract, infection with *A. schaalii* should be considered as a possible pathogen and correct microbiological investigations should be chosen accordingly (prolonged incubation time, different aerobic/anaerobic media, atmospheric conditions) [132,133,134,135,136,137,138,139,140,141,142,143,144,145,146,147]. Further studies are needed to evaluate additional risk factors and to define optimal choice and duration of antibiotic treatment [147].

## 5. Current and Emerging Treatment Options, Resistance

Antimicrobial therapy is the main treatment for genitourinary tract actinomycosis [1,12,37]. During the choice of therapy in *Actinomyces* infections, it is of pivotal for clinicians to be aware of antibiotics having relevant anti-anaerobe activity [145,146]. All anaerobic bacteria are resistant to the aminoglycosides, as the uptake of these antibiotics by bacterial cells occurs through oxygen- or nitrogen-dependent electron transport chains; this mechanism is uniformly in these pathogens, therefore, the drugs cannot reach their cellular targets. Similarly, fosfomycin, trimethoprim/sulfomethoxazole and most of the quinolones (with the exception of moxifloxacin, and novel, recently approved derivatives) have no or limited activity agains all anaerobic bacteria [1,12,37,148,149,150]. Non-spore forming Gram-positive rods are intrinsically resistant to metronidazole, due to their production of various enzymes, which inhibit the production of the reactive product responsible for the bactericidal effects of the drug [1,12,37,148,149,150]; this attribute makes these bacteria unique among anaerobes, as metronidazole resistance is otherwise very uncommon. Patients with genitourinary tract actinomycosis usually receive several weeks of intravenous high doses of a beta-lactam (i.e., penicillin G or V, ampicillin), followed by oral therapy (penicillin V or amoxicillin) in a sequential fashion for extended period of time (6–12 months) [1,12,37,148,149,150]. There are a number of reports detailing on the shorter (1–4 week) therapeutic regimens with successful clinical cure; however, there is currently insufficient evidence to safely warrant a change in current therapeutic guidelines [151,152]. In most cases, the administration of more broad-spectrum β-lactams (e.g., piperacillin-tazobactam, cefoxitin, ceftriaxone and carbapenems) is not required (except in the case of mixed infections with Gram-negative bacteria) as penicillin-derivatives result in adequate bactericidal effects [1,12,37,148,149,150,153]. In patients with penicillin-allergy, clindamycin may be successfully utilized [1,12,37,148,149,150]; clindamycin is also a potent anti-anaerobic agent, however, increased resistance-levels in other anaerobic genera (e.g., *Bacteroides*/*Parabacteroides* spp.) were noted [148].

As a general rule, routine antimicrobial susceptibility testing is not indicated for *Actinomyces* species and ALOs, and this is usually only performed in reference laboratories for surveillance purposes [148]. *Actinomyces* species have been described as being almost uniformly susceptible to most β-lactam antibiotics (>99% susceptibility to penicillin, amoxicillin, piperacillin-tazobactam, ceftriaxone, and carbapenems) and until recently, there were no concerns regarding the susceptibility of these microorganisms [148]. However, in the last decade, several reports have been published concerning the β-lactam resistance in *Actinomyces* spp. and ALOs. For example, recent publications have reported on *A. graevenitzii* and *A. europaeus* strains, which were resistant to ceftriaxone and piperacillin-tazobactam [154,155]; additionally, other Actinomyces speces were noted having high minimum inhibitory concentrations for cephalosporins and meropenem. In these strains, the underlying mechanisms of resistance was identified being the production of *bla*_TEM_-type β-lactamases and reduced membrane permeability [148,154,155]. During antimicrobial therapy of *Actinomyces* infections, recurrence is often observed; however, this may be attributed to other mechanisms instead of antimicrobial drug resistance. In the in vivo environments, many additional factors (e.g., protection of pathogenic bacteria by biofilm, poor pharmacokinetic profile of the anatomical region, inadequate tissue penetration of the antibiotic, poor circulation and presence of inflammation) have a role in therapeutic failure [148].

In addition to the administration of antibiotics, surgical procedures should also be included in the therapeutic protocols associated with *Actinomyces* infections, whether to provide an adjunctive role in the healing process (in milder cases) or to ensure the possibility of clinical success (reducing the formation of scar tissue, abscess drainage, excision of necrotic tissue and fistulas) [1,12,37,148,149,150,156]. Surgial intervention may have an additional benefit of reducing the duration and dose of antibiotic treatment required [1,12,37,148,149,150,156,157]. Removal of the IUDs is crucial in patients with IUD-associated actinomycosis [158]. Open surgical resection, often required for the definite diagnosis of genitourinary tract actinomycosis, facilitates the cure, but may be mutilating, especially if hysterectomy or bladder resection is performed [159]. There are no extensive data on the duration of antimicrobial therapy in such patients, but the duration of antimicrobials should probably be reduced in patients with extensive surgical resection of a small genital mass [1,12,37,148,149,150,156,157,158,159].

## Figures and Tables

**Table 1:** *Actinyomces* species with validly published names that have been implicated in human infections (based on [1,12,37]).

*A. bovis*	*A. israelii*	*A. radicidentis*
*A. cardiffiensis*	*A. massiliensis*	*A. radingae*
*A. dentalis*	*A. meyeri*	*A. timonensis*
*A. europaeus*	*A. naeslundii*	*A. turicensis*
*A. funkei*	*A. nasicola*	*A. urogenitalis*
*A. georgiae*	*A. neuii*	*A. viscous*
*A. gerencseriae*	*A. odontolyticus*	
*A. graevenitzii*	*A. oris*	
*A. hominis*	*A. oricola*	
*A. hongkongiensis*	*A. pyogenes*	

## Abbreviation

ABA	anaerobic blood agar
ALO	*Actinomyces*-like organism
ARDRA	amplified 16S rDNA restriction analysis
ARU	anaerobic reference unit
BBA	*Brucella* blood agar
BHI	brain-heart infusion (agar)
CFU	colony-forming unit
CT	computer tomography
G+C	guanine and cytosine content
HOMD	Human Oral Microbiome Database
ID	identification
IUD	intra-uterine contraceptice device
LPSN	List of Prokaryotic names with Standing in Nomenclature
LSA	lichen sclerosus et atrophicus
MALDI-TOF MS	matrix-assisted laser desorption/ionization time-of-flight mass spectrometry
MRI	magnetic resonance imaging
NGS	next-generation sequencing
PAS	Periodic acid–Schiff stain
PCR	polymerase chain reaction
PID	pelvic inflammatory disease
rRNA	ribosomal RNA
UTI	urinary tract infection
WGS	whole-genome sequencing
